# Soccer Player Characteristics in English Lower-League Development Programmes: The Relationships between Relative Age, Maturation, Anthropometry and Physical Fitness

**DOI:** 10.1371/journal.pone.0137238

**Published:** 2015-09-02

**Authors:** Ric Lovell, Chris Towlson, Guy Parkin, Matt Portas, Roel Vaeyens, Stephen Cobley

**Affiliations:** 1 School of Science and Health, University of Western Sydney, Penrith, Australia; 2 Department of Sport, Health and Exercise Science, The University of Hull, Hull, United Kingdom; 3 Pro-football Support, Huddersfield, United Kingdom; 4 Sport and Exercise Department, Teesside University, Middlesbrough, United Kingdom; 5 Department of Movement and Sports Sciences, Ghent University, Gent, Belgium; 6 Faculty of Health Sciences, The University of Sydney, Sydney, Australia; Universidad Europea de Madrid, SPAIN

## Abstract

The relative age effect (RAE) and its relationships with maturation, anthropometry, and physical performance characteristics were examined across a representative sample of English youth soccer development programmes. Birth dates of 1,212 players, chronologically age-grouped (i.e., U9’s-U18’s), representing 17 professional clubs (i.e., playing in Leagues 1 & 2) were obtained and categorised into relative age quartiles from the start of the selection year (Q1 = Sep-Nov; Q2 = Dec-Feb; Q3 = Mar-May; Q4 = Jun-Aug). Players were measured for somatic maturation and performed a battery of physical tests to determine aerobic fitness (Multi-Stage Fitness Test [MSFT]), Maximal Vertical Jump (MVJ), sprint (10 & 20m), and agility (T-Test) performance capabilities. Odds ratio’s (OR) revealed Q1 players were 5.3 times (95% confidence intervals [CI]: 4.08–6.83) more likely to be selected than Q4’s, with a particularly strong RAE bias observed in U9 (OR: 5.56) and U13-U16 squads (OR: 5.45–6.13). Multivariate statistical models identified few between quartile differences in anthropometric and fitness characteristics, and confirmed chronological age-group and estimated age at peak height velocity (APHV) as covariates. Assessment of practical significance using magnitude-based inferences demonstrated body size advantages in relatively older players (Q1 vs. Q4) that were *very-likely small* (Effect Size [ES]: 0.53–0.57), and *likely to very-likely moderate* (ES: 0.62–0.72) in U12 and U14 squads, respectively. Relatively older U12-U14 players also demonstrated small advantages in 10m (ES: 0.31–0.45) and 20m sprint performance (ES: 0.36–0.46). The data identify a strong RAE bias at the entry-point to English soccer developmental programmes. RAE was also stronger circa-PHV, and relatively older players demonstrated anaerobic performance advantages during the pubescent period. Talent selectors should consider motor function and maturation status assessments to avoid premature and unwarranted drop-out of soccer players within youth development programmes.

## Introduction

The relative age effect (RAE) is a well-established phenomenon in team-sports such as Soccer [1–4]. RAEs are characterised by an over-representation of players born earlier in their selection year, and are evident across the range of Soccer participation standards, including junior, youth representative, and professional levels [2,5]. Consequently, relatively older recreational and semi-professional players are afforded more playing opportunities [4], and having a relatively older squad in U17 national competitions has been associated with a higher final league ranking [6]. The underlying causes of the RAE in a Soccer context have not been distinguished empirically, nonetheless consensus implies that players born in the early months of the section year are likely at a physical advantage due to normative growth and/or biological maturation, and in the early stages of participation possess greater playing experience. This has been referred to as the maturation-selection hypothesis [2,7]. For the relatively younger, less biologically mature but equally motivated player, this may result in premature de-selection and drop-out. The growing concern for coaches and selectors is that skillful, but biologically delayed, soccer players may be lost in the early and developmental stages of athlete development.

Although the maturation-selection hypothesis has often been highlighted in pertinent literature (e.g., [8]), there have been few systematic attempts to explore the physical and anthropometrical advantages purported for relatively older players (e.g., [9,10]). An early consensus from these studies is that at the representative level, there are few differences in anthropometric and performance characteristics between players born in the first and last quartile of their annual age groups. Accordingly, it has been implied that coaches and talent selectors are biased towards players with advanced physical attributes, since elite-youth players born in the last three months of the selection year tend to be earlier maturers [11–13], enabling them to compete in absolute terms with their relatively older peers.

The origin for the existing data-sets available represents top-level European (e.g., [9]) and Asian [8] youth players, and the sampling populations typically represent an individual soccer club [8] or National Institution [9], with the exception of Deprez et al. [11,12] who’s samples were drawn from two elite Belgian academies. Conclusions from these studies make the assumption that data reflects the broader regional and cultural norm, including the diversity of playing styles and recruitment philosophies within or across broader soccer systems. However, Helsen et al. [2] showed that the proportion of U15-U18 national team players born in the first quartile of their selection year was higher in Germany and England (50%) versus Spain, the Netherlands, Belgium and Denmark (36–37%). Thus, it should be noted that criteria for talent identification and for onward player development may be specific as well as generic to given club(s), institution(s), national culture(s), and/or philosophies of its local and national coaching and scouting staff.

Another feature in existing research is that RAE magnitude (i.e., effect size between quartile 1 & 4) tends to decrease with advancing chronological age [9,14,15]. The highest discrepancies in quartile proportions have been associated with representative level sport in comparatively young age-cohorts (e.g. U6-U10, [14]). At this pre-adolescent stage, where the peak height velocity declines to its lowest rate since birth [16], the role of RAE in talent selection may be at its most influential. Thereafter, normative growth effects are superseded by the influence of biological maturation. The potential anthropometric and physical advantages afforded to relatively older players may be thus most pronounced at the earliest levels of talent selection. As this stage often represents the entry-point into athlete development pathways, any potential bias in talent selection could cascade through and across the developmental process as they gain access to more advanced coaching, competition, and facilities. To date, only Hirose [9] has sampled data from pre-adolescent soccer players at the entry-point of development programmes, they attempted to explore differences in body size, maturation, and physical fitness characteristics in players according to birth quartiles, but a shortage of quartile 4 players in the sample (i.e. U10: n = 2) prohibited this analysis. Consequently, the potential advantages for relatively older pre-adolescent players remain unexplored, and comprehensive insight of these relationships throughout the life-cycle of talent development systems has yet to be conducted in a substantive population of junior soccer players.

The aims of this study were twofold: 1) to examine the magnitude of the RAE in each annual age-group; and 2) to assess the relationships between relative age, maturation status, anthropometric and physical performance characteristics across the youth soccer development pathway. Specifically, we sought to determine the practical-significance of physical advantages afforded to relatively older players, in terms of anthropometry and physical fitness measures, and whether these changed across annual age-groups. Information of this nature is necessary to further understand the influences upon the talent selection process in representative youth soccer, and how these may vary across development stages. For this purpose, a broad sample of English Soccer Youth Squads across various age groups (i.e., U9-U18 players) was examined. To negate the impact of institutional-specific biases, and demonstrate representativeness, our sample was drawn from across 17 youth soccer academies, who themselves have established talent identification and development programmes, which resided within 17 English professional soccer clubs.

## Methods

### Participants

Following ethical approval (Department of Sport, Health and Exercise Science Ethics Committee, The University of Hull; Reference Number: 1415038), participants were *N* = 1,212 volunteering 9–18 year old soccer players, who within age-group categories (e.g., Under 16’s), were selected to represent one of 17 soccer clubs in their respective player development programmes. These clubs all had a professional adult team who competed in the professional lower divisions (i.e., League 1 & 2) of English Soccer at the time of data collection. The purpose of these youth-development programmes is to identify and nurture talented players to represent their club at the professional level. The U9 to U16 players trained 2–4 times per week after their school commitments, and played competitively in various game formats at the weekend, dependent upon their development stage. The U17 and U18 players were apprentices, with weekly training and match routines that were indicative of professional soccer schedules.

Informed consent was not specifically obtained from individual players for this study, instead this study was retrospective with data collected as part of routine monitoring processes administered by an external agency (Pro-Football Support) contracted by each club (approved by above named ethics committee). Players in these clubs were obliged to undertake the assessments as part of their contractual agreement.

That said, permission to use de-identified data was sought and provided by each individual football club for present study purposes.

### Procedures

#### Relative Age

Player birthdates were attained from club records and were categorised into birth quartiles (Q) within a specific age-category. In the UK, annual age-grouping is determined by the dates 1^st^ September- 31^st^ of August. Thus, Q1 players related to players born between September–November; Q2 = December–February; Q3 = March–May; and, Q4 = June–August.

All players in the sample also undertook a standardised battery of assessments to ascertain anthropometric attributes, maturation status, and physical fitness capacities. Players were familiarised with the procedures during the pre-season phase of the 2012/13 season, and the data used in this study was collected during the competitive phase of the season, specifically the months November-May at respective clubs. Assessments were administered by a small team of trained Sport and Exercise Science graduates, who had already gained 1–2 years of experience in delivering the test battery.

#### Anthropometrics

Height, sitting height (Harpenden Portable Stadiometer, Holtain, UK) and body mass (HD-366 Digital Weight Scale, Tanita, IL, US) were collected in accordance with procedures outlined previously [17]. Anthropometric data was collected in duplicate, with the average of the two measures recorded. Where height and body mass measures differed by more than 0.4 cm and 0.4 kg respectively, a third recording was taken, and the median value was assigned. Anthropometric data were used in conjunction with the players’ chronological age at the time of testing to estimate their somatic maturation using a regression equation developed by Mirwald et al. ([18]; equation 3). The equation derives an estimated maturity offset (years) from peak height velocity (PHV), from which the estimated age at PHV (APHV) was calculated. The coefficient of determination of the model was 0.89, with a 0.59 standard error of the estimate [18]. Using these procedures, the assessment team involved in the data collected demonstrated typical errors for intra- and inter-observer reliability of 0.20–0.23, and 0.19–0.31 years from PHV, respectively (determined as the standard deviation of the absolute differences, divided by the square root of 2). The Mirwald [18] technique has been used extensively (e.g. [19,20]), is a less invasive and more time efficient procedure relative to assessments of skeletal or sexual maturity stages, and was more convenient for the large multi-center cohorts in the current sample.

#### Physical Fitness

Aerobic capacity was determined via the Multi-Stage Fitness Test (MSFT; [21]). Players ran 20m shuttles at incremental running speeds until test termination. The test begins at 8.5 km/h^-1^ and increases by 0.5 km/h^-1^ at the end of each stage, which are approximately 1-min in duration (60-66s). Players either withdrew from the test voluntarily, or the test was terminated if the required running speed could not be sustained for two consecutive shuttles. Since maximal aerobic speed is underestimated by 2–3 km/h^-1^ with the MSFT when compared to track or treadmill-based incremental protocols [22] due to the MSFT’s repeated accelerations, decelerations, and turns, we used the distance covered (m) in the test as the outcome measure for aerobic capacity. Test-retest reliability of distance covered during the MSFT was determined in a subset of 44 U12-U16 players, and returned a typical error of 134 m (90% confidence intervals [CI]: 114–163 m).

Maximal Vertical Jump (MVJ) assessed players’ lower-limb explosive power. Counter-movement was permitted prior to upward propulsion, but arm-swing was restricted by clasping hips throughout the assessment. MVJ’s were performed on a contact mat (Just jump, Probotics, Inc. Huntsville, AL, USA) and trials were monitored visually to ensure minimal knee flexion upon landing. Players were permitted 2 practice attempts prior to the assessments, and then performed 5 MVJ’s separated by 60s recovery. MVJ height (cm) was taken as the average of the 3 highest jumps where scores differed by ≤2 cm. Where this criteria was violated, players performed additional trials (up to a maximum of 8) until the consistency requirement of the three highest jumps was satisfied.

Sprint performance was determined via three 20m maximal sprints. Players had a 1m rolling start and timing lights (Test Centre Timing System, Brower Timing Systems, Utah, USA) recorded the time taken to pass 10m and 20m markers. In between efforts, players walked for approximately 30s and recovered passively for a further two and a half minutes. The fastest time recorded for the sprints was used for analysis. The test-retest typical error for 10m and 20m sprint performance was 0.05 (90% CI: 0.04–0.06 s) and 0.08 s (90% CI: 0.07–0.10 s) respectively.

Agility was examined using the T-Test protocol described by Sporis et al. [23]. Players ran 9.14m forward and shuffled 18.28m between each side of the course, before running backwards for 9.14m to return to the starting position. Players performed the protocol on four occasions in total, leading the shuffling portion of the protocol with their right and left leg for two trials each to avoid bias according to limb-dominance. Each trial was separated by 2 minutes passive recovery. The fastest times leading with the right and left legs were averaged to determine agility performance.

### Statistical Analyses

*RAEs*: For each chronological age-group (U9-U18s), RAEs were determined using odds ratio’s (OR’s) and 95% confidence intervals (CI’s) for quartile and semester distributions. This technique contrasts relative age quartile frequency distributions against an expected equal distribution (i.e. 25 and 50% in each quartile and semester, respectively). National census data retrieved for UK births between 1993–2003 identified an approximately even birth distribution across quartiles (Q1: 25%; Q2: 24.2%; Q3: 25.1%; Q4: 25.7%) and semesters (1^st^ half: 49.2%; 2^nd^ Half: 50.2%) qualifying our expected frequency assumption. For the OR analyses, Q4 and S2 were assigned as referent groups.

#### Anthropometrics & Physical Fitness

Similar to Deprez et al. [12], to facilitate comparisons regarding anthropometric and physical characteristics according to birth quartile, we firstly categorised players into bi-annual age-groups (i.e., U10’s, U12’s, U14’s, U16’s, U18’s) due to the low frequency of relatively younger Q4’s in our representative player sample. Then initially, a one-way analysis of variance (ANOVA) determined the decimal age (DA) and maturation status (APHV) differences across relative age quartiles. Thereafter, a multivariate analysis of covariance (MANCOVA) assessed any anthropometric and physical performance differences between relative age quartiles. Chronological age-group (AG) and APHV were selected as covariates in the model, given their known influence on the dependent variables. Where main effects were identified, bonferroni post hoc analyses were preformed, with statistical significance assumed where *p* ≤ 0.05.

#### Practical Significance

Specific to the effect of relative age upon anthropometric and physical fitness measures, a magnitude based inferences approach [24] was utilised in a bid to isolate and identify findings that would be of most importance and value to practical application. *A priori*, data were log-transformed and the minimum practically important difference was defined as 0.2 between-subject standard deviations. Standardised thresholds for small (0.2), moderate (0.6) and large changes (1.2) were determined to examine the magnitude of differences in anthropometric and physical characteristics of Q1 versus Q4 players [24]. Mechanistic inferences were derived from the disposition of the confidence interval for the mean difference to these standardized thresholds. Using a customized spreadsheet [25], the probability that the true differences were substantial or trivial was calculated. Where the percent chances of Q1 versus Q4 differences were ≥ 5% in both a substantially positive and negative sense, the true effect was classified as unclear. In the event that a clear interpretation was possible, the following probabilistic terms were adopted: < 0.5%, *most unlikely*; 0.5–5%, *very unlikely*; 5–25%, unlikely; 25–75%, possibly; 75–95%, likely; 95–99.5%, very likely; > 99.5: most likely [24].

## Results

### RAEs

Relative age distributions between quartiles and semesters for each age-group are shown in Table 1. An un-even distribution was identified for each annual group, with 36–56% of players born in Q1 and 7–16% in Q4. For the entire cohort (i.e., across U9-U18’s), there was a 5.28 (95% CI’s: 4.08–6.83) greater chance of being enrolled into a player development programme if you were relatively older (Q1) versus being relatively (Q4) younger. Likewise, players born in the first semester of the annual-age group were 2.72 more likely to be represented than 2^nd^ Semester (95% CI’s: 2.22–3.34). The Q1 versus Q4 OR’s demonstrated a bi-modal distribution across advancing chronological age-groups. High over-representation of Q1 players was observed at U9 (OR: 5.56; 95% CI’s: 2.31–13.33) and progressively decreased until U12 (OR: 2.93; 95% CI’s: 1.36–6.35), thereafter OR’s were high between U13-U16 (5.45–6.13) and returned to lower levels at U17 (OR: 2.52; 95% CI’s: 1.26–5.06) and U18 (OR: 2.95; 95% CI’s: 1.42–6.10).

**Table 1:** Relative age distribution of junior playersin English Lower-League soccer clubs.

Age Category	Q1	Q2	Q3	Q4	N	Q1 v Q4 OR (CI)	Q2 v Q4 OR (CI)	Q3 v Q4 OR (CI)	1st v 2nd Half OR (CI)
All Players	48.6	24.6	17.7	9.2	1212	5.28 (4.08–6.83)*	2.67 (2.04–3.50)*	1.92 (1.46–2.54)*	2.72 (2.22–3.34)
U9	42.74	29.91	19.66	7.69	117	5.56 (2.31–13.33)*	3.89 (1.59–9.51)*	2.56 (1.01–6.45)*	2.66 (1.38–5.11)*
U10	51.64	18.85	18.85	10.66	122	4.85 (2.22–10.57)*	1.77 (0.76–4.12)	1.77 (0.76–4.12)	2.39 (1.27–4.51)*
U11	46.09	22.61	19.13	12.17	115	3.79 (1.73–8.29)*	1.86 (0.81–4.26)	1.57 (0.67–3.66)	2.19 (1.15–4.20)*
U12	36.07	32.79	18.85	12.30	122	2.93 (1.36–6.35)*	2.67 (1.23–5.80)*	1.53 (0.67–3.49)	2.21 (1.18–4.16)*
U13	46.15	22.31	23.08	8.46	130	5.45 (2.44–12.21)*	2.64 (1.13–6.15)*	2.73 (1.17–6.35)*	2.17 (1.18–4.00)*
U14	46.27	29.10	16.42	8.21	134	5.64 (2.53–12.55)*	3.55 (1.56–8.07)*	2.00 (0.84–4.76)	3.06 (1.65–5.69)*
U15	56.25	21.88	11.72	10.16	128	5.54 (2.57–11.93)*	2.15 (0.95–4.89)	1.15 (0.47–2.81)	3.57 (1.87–6.81)*
U16	53.85	21.98	15.38	8.79	91	6.13 (2.38–15.79)*	2.50 (0.92–6.83)	1.75 (0.62–4.98)	3.14 (1.48–6.66)*
U17	39.55	31.34	13.43	15.67	134	2.52 (1.26–5.06)*	2.00 (0.98–4.07)	0.86 (0.39–1.89)	2.44 (1.33–4.47)*
U18	47.06	22.69	14.29	15.97	119	2.95 (1.42–6.10)*	1.42 (0.65–3.09)	0.89 (0.39–2.05)	2.31 (1.21–4.38)*

Table Notes: Q1 = % of N (total players in category) born between Sept-Nov of annual age cut-off dates, Q2 = Dec-Feb, Q3 = March-May, Q4 = June-August; 1^st^ Half = % of N born between Sept-Feb; 2^nd^ Half = Mar-August; OR = Odds Ratio calculation, (CI) = 95% Confidence Interval.

* = Significant finding ≤ 0.05.

### Anthropometric Characteristics

MANCOVA revealed a higher body mass and stature in Q1 versus Q4 players in U10, U12 and U14, with U16 Q1 players also taller than Q4 (see Table 2). Age-group and APHV at the times of data collection were significant covariates for body mass and stature in each bi-annual age-group (*p* < 0.05). Assessment of practical significance identified that Q1 players had *very-likely small* advantages in stature and body mass versus Q4 players in U12 (Effect Size [ES]: 0.53–0.57), and *likely to very-likely moderate* advantages in U14 (ES: 0.62–0.72). *Unclear trivial-small* effects were identified between Q1 and Q4 players at the U10, U16 and U18 categories. ANOVA identified that APHV was younger in Q3 and Q4 when compared to Q1 at the U10 and U18 groups (*p* ≤ 0.01). No between quartile differences for APHV were observed for the U12, U14 and U16 groups.

**Table 2:** Decimal age, maturation, stature and body mass of junior soccer players according to relative age quartile & bi-annual age-groups.

Age Group	Variable	Q1	Q2	Q3	Q4	Covariates				F(Q)	*P*
						F(AG)	*P*	F(APHV)	*P*		
U10		*n = 86*	*n = 38*	*n = 23*	*n = 12*						
	DA	10.1 ± 0.5* ^	9.8 ± 0.6	9.8 ± 0.6	9.6 ± 0.6	-	-	-	-	5.39	0.001
	APHV	13.4 ± 0.4* ^#^	13.2 ± 0.4	13.1 ± 0.4	12.9 ± 0.5	-	-	-	-	8.43	0.000
	Stature (cm)	138.5 ± 5.9* ^#^	139.2 ± 4.0	139.9 ± 5.7	142.0 ± 6.2	75.34	0.000	129.11	0.000	4.65	0.004
	Body Mass (kg)	33.4 ± 4.3*	33.4 ± 3.6*	34.3 ± 4.6	34.5 ± 4.0	56.38	0.007	89.64	0.000	57.66	0.002
U12		*n = 74*	*n = 58*	*n = 35*	*n = 25*						
	DA	11.9 ± 0.5* ^#^	11.9 ± 0.5* ^#^	11.5 ± 0.5	11.2 ± 0.5	-	-	-	-	16.95	0.000
	APHV	14.0 ± 0.5	13.9 ± 0.4	13.8 ± 0.5	13.8 ± 0.3	-	-	-	-	2.06	0.106
	Stature (cm)	149.3 ± 7.7* ^#^ ^	150.4 ± 8.6*	147.5 ± 6.9	145.2 ± 6.3	257.43	0.000	315.86	0.000	18.24	0.000
	Body Mass (kg)	40.4 ± 6.7* ^#^ ^	40.8 ± 6.7*	38.2 ± 5.3	36.9 ± 4.8	158.22	0.000	285.82	0.000	19.14	0.000
U14		*n = 90*	*n = 48*	*n = 43*	*n = 16*						
	DA	13.9 ± 0.5* ^#^	13.8 ± 0.6* ^#^	13.4 ± 0.5	13.2 ± 0.5	-	-	-	-	14.52	0.000
	APHV	14.2 ± 0.6	14.1 ± 0.6	14.2 ± 0.6	14.1 ± 0.7	-	-	-	-	0.20	0.987
	Stature (cm)	164.2 ± 9.2*	163.8 ± 9.2*	160.5 ± 9.3*	158.8 ± 8.4	159.90	0.000	587.68	0.000	11.01	0.000
	Body Mass (kg)	52.3 ± 9.3*	52.5 ± 8.7*	49.7 ± 9.0*	48.2 ± 8.3	146.12	0.000	654.83	0.000	7.20	0.000
U16		*n = 90*	*n = 34*	*n = 20*	*n = 14*						
	DA	15.7 ± 0.4* ^#^ ^	15.5 ± 0.4*	15.3 ± 0.5*	14.9 ± 0.5	-	-	-	-	22.60	0.000
	APHV	14.2 ± 0.6	14.2 ± 0.6	14.1 ± 0.4	14.0 ± 0.6	-	-	-	-	0.876	0.455
	Stature (cm)	175.6 ± 6.3*	174.1 ± 7.0	175.3 ± 6.1	173.4 ± 8.1	30.52	0.000	158.69	0.000	3.79	0.012
	Body Mass (kg)	65.0 ± 7.2	62.6 ± 7.3	64.5 ± 7.1	65.1 ± 7.2	15.54	0.000	112.79	0.000	1.50	0.217
U18		*n = 91*	*n = 59*	*n = 31*	*n = 31*						
	DA	17.7 ± 0.5* ^#^ ^	17.4 ± 0.6*	17.1 ± 0.6	17.1 ± 0.6	-	-	-	-	17.64	0.000
	APHV	14.8 ± 0.6* ^#^	14.7 ± 0.6	14.5 ± 0.5	14.5 ± 0.5	-	-	-	-	3.93	0.009
	Stature (cm)	178.4 ± 6.3	179.4 ± 5.7	179.8 ± 5.8	178.6 ± 5.6	5.17	0.024	145.72	0.000	2.03	0.110
	Body Mass (kg)	73.0 ± 6.3	73.2 ± 7.2	72.9 ± 5.8	73.1 ± 6.8	4.30	0.039	78.15	0.000	1.80	0.147

Table Notes: Q1 = N players born between Sept-Nov of annual age cut-off dates, Q2 = Dec-Feb, Q3 = March-May, Q4 = June-August; DA = decimal age; AG = Chronological age-group; APHV = age at peak height velocity

* = Significant finding (P ≤ 0.05) vs. Q4

^#^ denotes significant vs. Q3

^^^ denotes significant vs. Q2

### Physical Fitness

MANCOVA identified few differences in physical fitness and performance attributes according to relative age quartile (see Table 3). Q3 (32.8 ± 7.2 cm) had a lower MVJ versus both Q1 (36.8 ± 6.2 cm) and Q4 players (36.8 ± 5.1 cm) in U18. The analysis of practical significance identified a number of anaerobic performance advantages in Q1 players (see Table 4). Q1 players compared to Q4’s had a *possibly small* agility (ES: 0.21–0.24) advantage in the U10 and U12 squads, and were *likely* faster over 10 (ES: 0.31–0.45; small effects) and 20 m (ES: 0.36–0.46: small effects) sprints in the U12 and U14 groups. Q1 players also had a greater MVJ performance versus Q4 in U14 (ES: 0.35; *small effect*). In contrast, a greater aerobic capacity was identified in Q4 players in U10 and U14 (ES: 0.23–0.31; *possibly small effects*). There were no between quartile differences in physical performance attributes denoted in the U16 and U18 squads.

**Table 3:** Fitness capacities of junior soccer players according to relative age quartile & bi-annual age-groups.

Age Group	Variable	Q1	Q2	Q3	Q4	Covariates				F(BQ)	P
						F(DA)	P	F(APHV)	P		
U10		*n = 86*	*n = 38*	*n = 23*	*n = 12*						
	MVJ	21.0 ± 4.4	19.9 ± 5.5	21.0 ± 4.4	20.3 ± 3.2	4.12	0.044	2.09	0.150	0.42	0.741
	MSFT	1180 ± 332*	1265 ± 259	1237 ± 315	1332 ± 306	16.20	0.000	0.09	0.765	3.92	0.010
	10m	1.96 ± 0.12	1.95 ± 0.09	1.95 ± 0.06	1.94 ± 0.07	4.80	0.030	0.03	0.865	1.37	0.253
	20m	3.61 ± 0.22	3.57 ± 0.18	3.57 ± 0.10	3.54 ± 0.14	4.89	0.029	0.04	0.836	1.87	0.136
	Agility	11.81 ± 0.77	11.80 ± 0.54	11.80 ± 0.60	11.75 ± 0.46	17.56	0.000	1.40	0.239	0.59	0.621
U12		*n = 74*	*n = 58*	*n = 35*	*n = 25*						
	MVJ	23.1 ± 6.5	24.3 ± 6.2	21.7 ± 5.4	22.5 ± 7.2	3.79	0.053	1.28	0.260	0.76	0.516
	MSFT	1456 ± 263	1481 ± 315	1354 ± 281	1441 ± 266	5.75	0.017	1.00	0.320	1.02	0.384
	10m	1.85 ± 0.09	1.85 ± 0.09	1.87 ± 0.06	1.89 ± 0.08	22.03	0.000	1.94	0.166	0.08	0.972
	20m	3.40 ± 0.18	3.38 ± 0.17	3.44 ± 0.10	3.48 ± 0.15	13.57	0.000	0.77	0.381	0.38	0.765
	Agility	11.02 ± 0.53	11.04 ± 0.56	11.28 ± 0.54	11.14 ± 0.58	16.14	0.000	1.40	0.239	0.99	0.401
U14		*n = 90*	*n = 48*	*n = 43*	*n = 16*						
	MVJ	27.9 ± 6.5	28.6 ± 5.8	26.8 ± 6.2	25.9 ± 6.9	23.87	0.000	3.18	0.076	0.33	0.804
	MSFT	1805 ± 285	1787 ± 280	1784 ± 374	1900 ± 242	10.01	0.002	0.42	0.519	1.69	0.170
	10m	1.71 ± 0.10	1.71 ± 0.09	1.74 ± 0.08	1.72 ± 0.07	44.62	0.000	32.92	0.000	0.87	0.460
	20m	3.12 ± 0.20	3.11 ± 0.16	3.17 ± 0.15	3.16 ± 0.15	52.27	0.000	33.30	0.000	0.79	0.503
	Agility	10.28 ± 0.61*	10.12 ± 0.47	10.36 ± 0.66	10.17 ± 0.41	42.18	0.000	5.46	0.021	3.18	0.025
U16		*n = 90*	*n = 34*	*n = 20*	*n = 14*						
	MVJ	28.2 ± 8.1	26.9 ± 8.4	30.7 ± 7.0	26.9 ± 8.5	0.19	0.665	3.24	0.074	1.10	0.351
	MSFT	2085 ± 257	2170 ± 280	2083 ± 295	1976 ± 311	8.71	0.004	0.22	0.638	1.80	0.150
	10m	1.61 ± 0.08	1.61 ± 0.07	1.63 ± 0.11	1.63 ± 0.09	3.57	0.061	4.13	0.044	0.27	0.848
	20m	2.92 ± 0.12	2.91 ± 0.13	2.95 ± 0.20	2.94 ± 0.17	5.70	0.018	3.12	0.079	0.55	0.649
	Agility	9.63 ± 0.48	9.60 ± 0.51	9.61 ± 0.53	9.80 ± 0.58	6.38	0.013	4.37	0.038	0.63	0.599
U18		*n = 91*	*n = 59*	*n = 31*	*n = 31*						
	MVJ	36.8 ± 6.2^#^	35.2 ± 4.4	32.8 ± 7.2	36.8 ± 5.1^#^	2.82	0.094	4.30	0.039	4.25	0.006
	MSFT	2308 ± 285	2270 ± 277	2308 ± 270	2325 ± 259	1.17	0.281	4.74	0.031	0.39	0.763
	10m	1.59 ± 0.06	1.60 ± 0.05	1.61 ± 0.04	1.58 ± 0.06	1.68	0.197	0.98	0.323	1.29	0.280
	20m	2.87 ± 0.11	2.87 ± 0.08	2.90 ± 0.08	2.85 ± 0.10	0.39	0.534	1.00	0.318	1.37	0.532
	Agility	9.20 ± 0.40^	9.42 ± 0.55	9.32 ± 0.36	9.29 ± 0.38	0.00	0.989	1.43	0.234	2.56	0.056

Table Notes: Q1 = N players born between Sept-Nov of annual age cut-off dates, Q2 = Dec-Feb, Q3 = March-May, Q4 = June-August; DA = decimal age; AG = Chronological age-group; MVJ = maximal vertical jump; MSFT = multi-stage fitness test; 10m and 20m = sprint times over stated distances

* = Significant finding (P ≤ 0.05) vs. Q4

^#^ denotes significant vs. Q3

^^^ denotes significant vs. Q2

**Table 4:** Practical significance assessment of anthropometric & fitness capacities between relatively older and younger junior soccer players according to bi-annual age-groups.

Age Group	Variable	Q1	Q4	Mean Diff % (± 90% CI)	ES	Magnitude	Qualitative
U10		*n = 95*	*n = 17*				
	Stature	137.8 ± 6.0	139.1 ± 7.0	0.9 ± 2.1	0.21	Small	Unclear
	Body Mass	33.1 ± 4.5	32.7 ± 4.2	1.2 ± 5.3	-0.09	Trivial	Unclear
	MVJ	20.6 ± 4.6	19.8 ± 3.7	-3.2 ± 8.3	-0.14	Trivial	Unclear
	MSFT	1160 ± 326	1229 ± 304	7.0 ± 11.6	0.23	Small	Possibly
	10 m Sprint	1.96 ± 0.11	1.97 ± 0.09	0.5 ± 2.1	0.08	Trivial	Unclear
	20 m Sprint	3.61 ± 0.22	3.60 ± 0.16	-0.2 ± 2.1	-0.04	Trivial	Unclear
	Agility	11.86 ± 0.76	12.04 ± 0.73	1.6 ± 2.7	0.24	Small	Possibly
U12		*n = 88*	*n = 28*				
	Stature	149.5 ± 7.7	145.1 ± 6.0	-2.9 ± 1.5	-0.57	Small	Very Likely
	Body Mass	40.4 ± 6.6	36.9 ± 4.6	-8.3 ± 4.4	-0.53	Small	Very Likely
	MVJ	23.1 ± 6.4	22.4 ± 7.1	-5.9 ± 11.8	-0.22	Small	Unclear
	MSFT	1468 ± 263	1430 ± 266	-2.7 ± 6.9	-0.14	Trivial	Unclear
	10 m Sprint	1.85 ± 0.09	1.89 ± 0.08	2.2 ± 1.6	0.45	Small	Likely
	20 m Sprint	3.40 ± 0.17	3.48 ± 0.14	2.4 ± 1.6	0.46	Small	Likely
	Agility	11.03 ± 0.54	11.14 ± 0.57	1.0 ± 1.9	0.21	Small	Possibly
U14		*n = 102*	*n = 19*				
	Stature	164.4 ± 8.9	158.0 ± 8.3	-3.9 ± 2.2	-0.72	Moderate	Very Likely
	Body Mass	52.3 ± 8.9	47.0 ± 8.4	-10.2 ± 6.9	-0.62	Moderate	Likely
	MVJ	27.6 ± 6.5	25.6 ± 7.4	-8.7 ± 12.6	-0.35	Small	Possibly
	MSFT	1794 ± 293	1878 ± 236	5.3 ± 6.1	0.31	Small	Possibly
	10 m Sprint	1.71 ± 0.10	1.74 ± 0.08	1.8 ± 2.1	0.31	Small	Possibly
	20 m Sprint	3.12 ± 0.19	3.19 ± 0.17	2.2 ± 2.3	0.36	Small	Likely
	Agility	10.26 ± 0.60	10.31 ± 0.57	0.5 ± 2.4	0.08	Trivial	Unclear
U16		*n = 107*	*n = 17*				
	Stature	175.5 ± 6.5	173.8 ± 8.2	-1.0 ± 2.1	-0.27	Small	Unclear
	Body Mass	65.1 ± 7.4	64.9 ± 6.8	-0.1 ± 4.7	-0.01	Trivial	Unclear
	MVJ	27.0 ± 8.3	26.5 ± 8.0	-1.1 ± -14.0	-0.03	Trivial	Unclear
	MSFT	2090 ± 2262	2003 ± 317	-4.5 ± 7.0	-0.37	Small	Unclear
	10 m Sprint	1.61 ± 0.07	1.62 ± 0.09	0.6 ± 2.4	0.14	Trivial	Unclear
	20 m Sprint	2.91 ± 0.12	2.91 ± 0.17	0.0 ± 2.6	-0.01	Trivial	Unclear
	Agility	9.64 ± 0.47	9.74 ± 0.61	1.0 ± 2.7	0.21	Small	Unclear
U18		*n = 106*	*n = 38*				
	Stature	179.0 ± 6.9	178.8 ± 6.2	-0.1 ± 1.1	-0.02	Trivial	Unclear
	Body Mass	73.5 ± 6.7	73.0 ± 6.5	-0.7 ± 2.8	-0.07	Trivial	Unclear
	MVJ	36.8 ± 6.0	36.2 ± 5.0	-1.3 ± 4.5	-0.08	Trivial	Unclear
	MSFT	2304 ± 287	2324 ± 253	1.0 ± 3.9	0.08	Trivial	Unclear
	10 m Sprint	1.59 ± 0.07	1.58 ± 0.06	-0.8 ± 1.3	-0.19	Trivial	Possibly
	20 m Sprint	2.87 ± 0.11	2.85 ± 0.09	-0.5 ± 1.1	-0.13	Trivial	Possibly
	Agility	9.22 ± 0.41	9.26 ± 0.39	0.5 ± 1.4	0.10	Trivial	Unclear

Table Notes: Q1 = N players born between Sept-Nov of annual age cut-off dates, Q4 = June-August; ES = effect size; MVJ = maximal vertical jump; MSFT = multi-stage fitness test.

## Discussion

In this study, our aim was to firstly quantify the magnitude of the relative age effect in each annual age-category spanning the entire cycle of player development at the representative level in English soccer. In this regard, findings illustrate that relatively older players (quartile 1) of their selection year were 5.3 times more likely to be registered and participating in development programmes compared to their quartile four (relatively younger) contemporaries, with the bias most pronounced at U9 and U13-U16. Odds ratios were equivocal with a broader study of English youth-representative players [3], but larger than those determined in Belgian soccer (OR’s: 0.7–3.6; [14,15]), suggesting that a substantially large RAE is present across and within the youth departments of English lower-league professional clubs.

Our second aim was to examine the relationships between relative age, maturation, anthropometry and physical fitness characteristics, and in particular focus on how these relationships varied across the different stages of youth soccer development. We assessed whether any physical or performance advantages were conferred to relatively older players, and by what likely mechanisms, via null-hypothesis significance testing, with a statistical model that recognized the confounding influence of chronological age-group and maturation upon the dependent variables (i.e., anthropometric and fitness characteristics). Analyses revealed similar findings to previous research [8,9,12] demonstrating that relatively older players had no physical advantages in representative youth-soccer, leading to the conclusion that the few selected relatively younger players were as biologically advanced so as to equally compete physically with their relatively older team-mates.

In practice, the talent selector and coach cannot always use advanced statistical models and account for confounding variables in their selection policies. Therefore, we also adopted the approach of Deprez and colleagues [12], by quantifying the practical relevance of anthropometric and performance differences owing to RAE, via the magnitude-based inferences technique [24]. This approach evaluates the effect magnitude in reference to the smallest practically beneficial or harmful values, and is applicable to the decision-making process of the selector and/or coach. Accordingly, interpretation of our results is founded upon the assessment of practical significance, of which our most important findings include: a) Q1 players had height and weight advantages versus Q4 in the U12 and U14 age-groups; b) Q1 players demonstrated greater anaerobic performances (agility, speed and MVJ) in younger annual age-groups (U10-U14); and c) The influence of relative age upon anthropometric or performance characteristics was absent in U16-U18 groups. As the relationships between relative age, maturation status, anthropometric and physical performance characteristics varied according to annual age-groupings, we now discuss these findings in chronological order.

### Under 9’s & 10’s

RAEs were notably high at the entry point of talent development programs (i.e., U9), with Q4 players 5.6 times less likely to be registered and participating compared to Q1 born players. To our knowledge, the only equivalent comparative data is by Helsen et al. [15], who reported a lower effect magnitude in U10-12 Belgian players (OR: 1.8–2.2). The high RAE in our data suggests that the anthropometric and physical developmental differences between the relatively older and younger are most pronounced at this stage. While age-group was a significant covariate in the MANCOVA, no practical anthropometric advantages for the relatively older players were apparent, but when benchmarking players according to UK growth charts [26], findings revealed that the stature of the relatively younger U10 players (Q4) were between the 75th and 91st centile, whereas Q1 players resided around the 50th centile for their chronological age. Hence, relatively younger players were not disadvantaged in an anthropometric sense at this age early stage, but were likely selected due to their advanced normative growth, which was more matching and befitting to a relatively older player.

In terms of physical fitness, relatively older players at this stage did demonstrate superior agility performance and had a slightly weaker MSFT performance, although both were of only *possibly small* practical significance. The anthropometric and fitness differences between Q1 and Q4 might again be explained by the more advanced growth status of selected Q4 players. Q4 players in the sample did have a younger estimated APHV when compared to their relatively older team-mates. Taken together, this suggests that players at these ages demonstrated a homogenous anthropometric and fitness phenotype, whereby only relatively younger players with advanced normative growth and/or maturation, were nominated to receive advanced coaching opportunities. Alternatively, the lack of anthropometrical and physical differences between Q1 and Q4 players might imply that the strong RAE observed in pre-adolescent squads may be caused by factors beyond the scope of this study, such as the potential experience and skill advantages afforded to relatively older players.

### Under 11’s & 12’s

From Under 9–12’s, a steady decline in the RAE magnitude was observed and characterised by a small frequency increase in the number of Q4 players (i.e., 7.7–12.3%). The accessibility of relatively younger players to development programmes remained however, and such players were still disadvantaged in terms of stature and body mass. Normative growth declines to its lowest rate since birth at approximately 11 years of age [16], but then begins a rapid acceleration towards peak height velocity from circa-12 years. Since the estimated age at peak height velocity of the U11-U12 players was not different between birth quartiles, the anthropometric differences observed likely reflect the relatively older players earlier onset of the adolescent growth spurt. Relatively older players in these squads were also afforded *likely small* physical advantages in terms of sprint and agility performance, which may be an important underlying factor in the RAE given the value of speed qualities in talent selection outcomes [27] and goal situations [28].

### Under 13’s & 14’s

Greater magnitudes of anthropometric advantages for Q1’s were observed in the *Under 13’s & 14’s*, coinciding with the approach to peak height velocity during maturation. These distinct advantages for the relatively older players during the onset of the pubescent growth spurt are also likely to contribute to the increasing RAEs observed from this point onward until *Under 16’s*. Bi-annual age-groups showed small speed (i.e., 10 & 20m sprint) and power (i.e. MVJ) advantages were afforded to relatively older players, which may contribute to RAE bias during the pre to circa-PHV transition. The precise underlying mechanism for greater sprint and jump capacity at this stage is not known, but may relate to a range of biological, neurological and biomechanical factors that inter-play during maturation. Given the greater stature and body mass of relatively older players, it is tempting to speculate that increased muscle length and cross-sectional area could be accountable [29]. Though, longitudinal research in 11–13 year-old physical education students identified no association between growth rates and improvements in running speed [30], and Medez-Villanueva et al. [31] identified that age-related enhancements in sprint running performances were almost exclusively related to differences in maturation rather than anthropometric factors *per se*. In our sample, relatively older players at this stage were generally in the early phases of the adolescent maturation spurt, and so these players may benefit from enhanced neural function, co-ordination, muscle architecture, and hormonal-induced increases in muscle power [32]. Irrespective of the underlying mechanisms, the mean difference in sprint times between Q1 and Q4 players (1.8–2.4%; small effect sizes) was of a similar magnitude to that identified between retained and drop-out players from youth soccer development programs in Belgium (2.1–3.3%, small-moderate effect sizes; [27]). Thus, without due consideration for maturation status, based on absolute performances in physical assessments, training, and matches, the faster and more powerful relatively older Q1 players are more likely to be enrolled and retained in development programmes.

### Under 15’s & 16’s

At this stage, RAEs remained high (OR: 5.54–6.13). However, there were no practically significant differences in anthropometric, maturation status, and physical characteristics between Q1 and Q4 players. RAEs here may now be indicative of a “cascade” effect, and the lagging of anthropometric and physical advantages from earlier adolescent growth spurts that occur during prior age-groups.

### Under 17’s & 18’s

The minimal anthropometric and physical differences between the relatively older and younger players were also apparent in these squads (see Table 4). Also, these latter age-groups were also categorised by a marked reduction in RAE magnitude (OR: 2.52–2.95) when compared to *Under 9’s & 10’s* and *Under 13’s-16’s*. Similar reductions in representative level soccer players were observed by Helsen et al. [15] and again can be explained by the transient nature of the advantages afforded to relatively older or advanced maturers during puberty, while relatively younger players “catch-up” by going through their final growth spurt at a later stage. What is not clear, is how more relatively young players enter or return to the developmental pathway at *Under 17’s & 18’s*, and future research is warranted to determine how RAE bias at the entry-point of the developmental pathway influences selection policy at a later stage, and how non- or de-selected players enter or return to these squads.

To our knowledge, this is the first study to examine the maturation-selection hypothesis across the entire soccer development pathway in a substantive population. The RAE was particularly strong at the entry-point of the representative-level soccer, and again during puberty, conferring anthropometric and anaerobic performance advantages to U11 to U14s, whereas relative age had little or no effect on players’ endurance capacity. These trends at the entry-point of the development pathway represent a significant challenge for non-registered or relatively younger players whom are less biologically mature. These players may not receive the same exposure to advanced coaching and training facilities, or may even result in drop-out [1,14]. To ensure that equally talented, but relatively younger players, are not de-selected prematurely based on physical attributes, coaches and talent selectors may consider evaluating each players ability based on individual technical performance assessments [12,33], rather than relying exclusively on competitive game-formats in which their combative physical features favor pre-adolescent players that are relatively older, or advanced maturers.

The main limitations in the current study relate to the cross-sectional nature of the data-set, which constrain the ability to offer causal explanation; the focused measurement and assessment of anthropometric and fitness parameters, and the non-invasive estimations of somatic maturation within such measures. We recognize that the Mirwald [18] maturation estimation underestimates APHV in males under the age of 12, and over-estimates APHV in males over the age of 13 [34]. This limitation is evidenced in the current study, as the mean values of APHV increase with chronological age (see Table 2). Accordingly, the higher than average mean values for APHV in the oldest age groups reflect a limitation in the method for assessing biological maturation, and not a selection bias towards late maturing males. However, we employed the estimation procedure as a covariate and a dependent variable in a cross-sectional manner to examine the RAE in discreet chronological age-groups. Accordingly, the confounding influence of chronological age on the longitudinal APHV estimation [34] was somewhat irrelevant in our study design. As the Mirwald procedure [18] provides a practical, non-invasive tool for large multi-site data collection (e.g., see [19,20]), we accepted and traded its limitations for the purposes of obtaining an extensive representative sample in the English youth soccer context. Finally, while physical fitness measures were prioritized in this study, the assessment of relationships with psycho-social, environmental, and technical/tactical factors remain valuable future directions.

In summary, our findings identified a strong relative age effect bias in the selection of representative-level squads in the youth divisions of lower-league English Soccer clubs. This bias was particularly strong at the entry-point to the developmental pathway (U9) and during the pubescent growth spurt (U13-U16). Relatively younger players selected were typically advanced for their chronological age in terms of maturation status and anthropometric characteristics. Circa-PHV, we observed small-moderate advantages in body size and anaerobic performance capacities in relatively older players (U11-U14), but these were transient and not observed in U15-U18. Collectively, findings suggest that coaches and talent identification and development practitioners operating in this demographic favour players with advanced maturation and physical capacities, which may limit the opportunities of the equally skilled and motivated players but who are less biologically mature. Given the dynamic nature of the talent development process, practitioners should routinely track the progress of individual motor function, maturation status, as well as performance characteristics to inform talent identification and retention policies in youth development programmes, and to avoid premature de-selection and drop-out of talented soccer players.
